# Comment on: “Handgrip weakness, low fat‐free mass, and overall survival in nonsmall cell lung cancer treated with curative‐intent radiotherapy" by Burtin et al.

**DOI:** 10.1002/jcsm.12637

**Published:** 2020-12-19

**Authors:** Azadeh Shafieesabet, Wolfram Doehner

**Affiliations:** ^1^ Berlin Institute of Health Center for Regenerative Therapies (BCRT), Department of Cardiology (Virchow Klinikum) Charité—Universitätsmedizin Berlin, German Centre for Cardiovascular Research (DZHK), partner site Berlin Berlin Germany; ^2^ Center for Stroke Research Berlin (CSB) Charité—Universitätsmedizin Berlin Berlin Germany

We read with great interest the article by Burtin *et al*.^1^ This study has shown that handgrip weakness and low fat‐free mass index (FFMI) are independent prognostic factors for overall survival (OS) in patients with early and locally advanced non‐small cell lung cancer (NSCLC) treated with curative‐intent (chemo) radiation.

In the current study, handgrip weakness is defined as maximal handgrip strength below the 10th percentile of the UK Biobank reference values, taking gender, age, and height into account in at least one side, while in this reference percentile 5 was calculated as a point of reference for abnormally low handgrip strength.^2^

Cancer cachexia is a multifactorial syndrome characterized by a progressive loss of skeletal muscle mass, along with adipose tissue wasting, systemic inflammation, and other metabolic abnormalities leading to functional impairment.^3^, ^4^, ^5^ Cancer cachexia has long been recognized as a direct cause of complications in cancer patients, reducing quality of life and worsening disease outcomes.^6^ Although cancer cachexia is characterized by other factors like anorexia, fatigue, and loss of muscle strength,^7^ body weight (BW) loss is one of the core factors of cancer cachexia, which is consistent with the criteria proposed by Fearon or Evans that implicate BW loss as a key factor in cancer cachexia.^8^, ^9^

Mytelka *et al*. reported that increasing weight loss led to substantially worse outcomes for NSCLC patients independent of other variables.^10^ Similarly, Takayama *et al*. demonstrated that BW loss most likely shortened survival in patients with advanced NSCLC and should be closely monitored.^7^ These results suggest the important role of cachexia in prognosing the survival in advanced NSCLC patients.

Burtin *et al*. demonstrated that handgrip weakness (hazard ratio = 1.31, 95% confidence interval: 1.07–1.59, *P* = 0.008) and low FFMI (hazard ratio = 1.24, 95% confidence interval: 1.03–1.51, *P* = 0.024) were associated with OS.^1^ Thus, because the model is adjusted only for age, gender, disease stage, and Charlson comorbidity index, it is unable to show any impact of weight loss, body mass index, or cachexia on survival. Furthermore, low FFM was defined as an FFMI below 17 kg/m^2^ in male patients and below 15 kg/m^2^ in female patients, in line with the Global Leadership Initiative on Malnutrition (GLIM) criteria for the diagnosis of malnutrition. However, it is recommended to use the GLIM consensus criteria for malnutrition in parallel with established concepts, including cachexia or sarcopenia.^11^ We suggest that baseline body mass index and weight loss should be included in the model to provide a precise approach for identifying poor prognosis in NSCLC patients.

## Conflict of interest

The authors declare that they have no conflict of interests and certify that they comply with the ethical guidelines for authorship and publishing in the Journal of Cachexia, Sarcopenia and Muscle.^12^
